# Outcome measurement in clinical trials for Ulcerative Colitis: towards standardisation

**DOI:** 10.1186/1745-6215-8-17

**Published:** 2007-06-25

**Authors:** Rachel M Cooney, Bryan F Warren, Douglas G Altman, Maria T Abreu, Simon PL Travis

**Affiliations:** 1 Gastroenterology Unit, John Radcliffe Hospital, Oxford, OX3 9DU, UK; 2 Centre for Statistics in Medicine, University of Oxford, Oxford, UK; 3 Inflammatory Bowel Disease Center, Mount Sinai Hospital, Division of Gastroenterology, New York, New York USA

## Abstract

Clinical trials on novel drug therapies require clear criteria for patient selection and agreed definitions of disease remission. This principle has been successfully applied in the field of rheumatology where agreed disease scoring systems have allowed multi-centre collaborations and facilitated audit across treatment centres. Unfortunately in ulcerative colitis this consensus is lacking. Thirteen scoring systems have been developed but none have been properly validated. Most trials choose different endpoints and activity indices, making comparison of results from different trials extremely difficult. International consensus on endoscopic, clinical and histological scoring systems is essential as these are the key components used to determine entry criteria and outcome measurements in clinical trials on ulcerative colitis. With multiple new therapies under development, there is a pressing need for consensus to be reached.

## Background

Clinical trials determining the efficacy of new treatments need internationally agreed standardised endpoints. Only these allow studies to be compared and, importantly, combined for greater statistical power and a more reliable estimate of the benefits and harms of an intervention. Agreement on endpoints has been achieved for trials in rheumatology (Outcome Measures in Rheumatology, OMERACT [1]. OMERACT's consensual approach has been extremely successful and we feel that this approach now needs to be applied to trials of inflammatory bowel disease.

In the field of gastroenterology there are many exciting new drugs in development, with great prospects for the treatment of ulcerative colitis in particular. International consensus on the endoscopic, clinical and histological scoring systems is essential as these are the key components used to determine entry criteria and outcome measurements in clinical trials of ulcerative colitis. As the aim of all clinical trials is to determine whether an intervention results in clinical response and/or remission, with an acceptable adverse event profile, an agreed definition of these parameters is paramount. Whereas in rheumatology joint space narrowing may be measured with simple radiography, in inflammatory bowel disease direct measures are more difficult and often involve endoscopy. In this paper we discuss the systems currently available and their limitations. We propose potential solutions, focussing in particular on the issue of interobserver variation in sigmoidoscopy.

### Clinical scores

The multiplicity of clinical activity indices used for scoring ulcerative colitis has recently been comprehensively reviewed [2]. No less than seven different symptom-based activity scores, two composite scores, and four evaluation scoring systems have been used in ulcerative colitis [3-16] [Table 1]. The names of the indices also vary between different publications which exacerbates the confusion [Table 2]. The scores vary in the use of objective measurements (stool frequency, temperature, pulse rate, results of blood tests), subjective components (physician's global assessment, general well being), and sigmoidoscopy, which is itself open to wide inter-observer variation [Table 3].

**Table 1:** Summary of activity indices used for ulcerative colitis

**Index**	**Year**	**Also known as**	**References**
**Clinical/biomedical**			
Truelove &Witts'	1955		3
Powell Tuck	1978	St Mark's Index	4
Rachmilewitz	1988	Clinical Activity Index (CAI)	7
Lichtiger	1990	Modified Truelove &Witts' Severity Index	8
Seo	1992	Activity index (AI)	9
Walmsley	1998	Simple Clinical Colitis Index (SCCAI)	10
Feagan	2005	Ulcerative Colitis Clinical Score (UCCS)	14

**Composite **(clinical and endoscopic)			
Schroeder	1987	Mayo score, Disease Activity Index (DAI)	5
Sutherland	1987	Ulcerative Colitis Disease Activity Index (UCDAI)	6

**Evaluation**			
Physician's Global Evaluation	1993	PGA	11
Investigator's Global Evaluation	1998		12
Individual Symptom Score	2002		13
Patient Defined Remission	2005		19

**Quality of life**			
Inflammatory Bowel Disease Questionnaire		IBDQ	15
Short-form 36		SF36	39

**Table 2:** Summary of different properties measured in clinical activity indices

	**Author and name of index**						
	**Truelove &Witts **[3]	**Powell-Tuck **[4]	**Schroeder **[5]	**Rachmilewitz **[7]	**Lichtiger **[8]	**Seo **[9]	**Walmsley **[10]

**Property**		St Mark's index	Mayo score, Disease Activity Index (DAI)	Clinical Activity Index (CAI)	Modified Truelove &Witts' Severity Index (MTWSI)	Activity Index	Simple Clinical Colitis Index (SCCAI)

Score range	Mild, Moderate, Severe	0–24	0–12	0–23	0–21	70–300	0–20
Bowel frequency/24 hrs	<4 (mild) >6 (severe)	0–2 (<3 to >6)	0–3 (normal to ≥ 5 above normal)	0–3(<18 to >60/week)	0–4 (≤ 2 to ≥ 10)	add total number/24 hr × 13	0–3(day) 1–2(night)
Stool form		0–2			Need for antidiarrhoeal agents		
Urgency					Faecal incontinence 0 or 1		0–3
Blood		0–2	0–3	0–4	0–3	add bloody stools/24 hr × 60	0–3
Abdominal Pain		0–2		0–3	0–3		0–4
Abdominal tenderness		0–4					
General well-being		0–3		0–3	0–5		0–4
Anorexia		0–1					
Nausea/Vomiting		0–1					
Extra-intestinal features		0–3		0–9			0–5
Fever	>37.5°C (severe)	0 – 2 (<37.1 to >38°C)		0 (<38°C) or 3 (>38°C)			
Tachycardia	>90						
Anaemia	<75% of normal			0 or 4		subtract value × 4	
ESR mm/hr	>30			0–2		add value × 0.5	
Albumin						subtract value × 15	
Sigmoidoscopy		0–2	0–3	0–12			
Global assessment			0–3	0–3	0–5	Score = sum of the above and add 200	

Note: precise details of the individual components of each score are too complex to be included in a single table: refer to original article or ref [2]

**Table 3:** Endoscopic scores for ulcerative colitis

**Score [ref]**	**0**	**1**	**2**	**3**	**4**
Baron [ 21]	Normal: matt mucosa, ramifying vascular patter clearly visible, no spontaneous bleeding, no bleeding to light touch	Abnormal, but non-haemorrhagic: appearances between 0 and 2	Moderately haemorrhagic: bleeding to light touch, but no spontaneous bleeding seen ahead of the instrument on initial inspection	Severely haemorrhagic: spontaneous bleeding seen ahead of instrument at initial inspection and bleeds to light touch	-
Powell-Tuck [4]	Non-haemorrhagic (no bleeding spontaneously or on light touch)	Haemorrhagic (bleeding on light touch, but no spontaneous bleeding ahead of instrument)	Haemorrhagic (spontaneous bleeding seen ahead of instrument on initial inspection and bleeding to light touch)	-	-
Rachmilewitz [7]	No granulation scattering light, normal vascular pattern, no mucosal vulnerability, no mucosal damage (mucus, fibrin, exudates, ulcer)	Faded or disturbed vascular pattern	Granulation scattering light, completely absent vascular pattern, contact bleeding, slight mucosal damage	Spontaneous bleeding,, pronounced mucosal damage	-
Lémann [16]	Normal mucosa	Oedema and/or loss of mucosal vascularity, granularity	Friability (visible, induced bleeding on examination), petechiae	Spontaneous haemorrhage, visible ulcers	-
Schroeder [5]	Normal or inactive disease	Mild (erythema, decreased vascular pattern, mild friability)	Moderate (marked erythema, absent vascular pattern, friability, erosions)	Severe (spontaneous bleeding, ulceration)	-
Sutherland [6]	Normal	Mild friability	Moderate friability	Exudation, spontaneous haemorrhage	-
Feagan [14]	Normal, smooth, glistening mucosa, with vascular pattern visible; not friable	Granular mucosa; vascular pattern not visible; not friable; hyperaemia	As 1, with a friable mucosa, but not spontaneously bleeding	As 2, but mucosa spontaneously bleeding	As 3, but clear ulceration; denuded mucosa

In 1955 Truelove and Witts [3] were the first to attempt to quantify disease activity defining mild, moderate and severe disease. Endoscopy was added into a continuous scale developed by Powell-Tuck and colleagues [4]. In the early 1980s the eleven components of this index were simplified in the Mayo score [5] and the Ulcerative Colitis Disease Activity Index (UCDAI or Sutherland Index) [6], which have three clinical variables and an endoscopy score. Later, Rachmilewitz proposed the Clinical Activity Index (CAI) which includes laboratory data as well as clinical and endoscopic variables [7]. Other non-invasive indices have been developed including the Seo index [9] which measures symptoms and some simple laboratory values (haemoglobin, erythrocyte sedimentation rate and albumin) and the simple clinical colitis activity index (SCCAI) [10] which has six clinical questions only. However, none of these scoring systems has been validated with a formal evaluation of their biometric properties (responsiveness, reliability and validity) [17].

Scoring systems for ulcerative colitis are driven by the need to select appropriate patients and monitor response in clinical trials, which is why interest waxes in time with drug development (steroids in the 1950s, sulfasalazine in the next two decades, mesalazine in the 1980s and ciclosporin in the 1990s). Now, in 2007, there are up to 30 new agents being evaluated for the treatment of ulcerative colitis in phase 2 and 3 trials [18], and there has been a resurgence of interest in scoring systems. Yet only one system (Truelove and Witts' [3]) is simple and objective enough to use in clinical practice, as well as aiding clinical decision making, but this score suffers from a lack of responsiveness to changes in symptoms following an intervention. Consequently the Food and Drug Administration (FDA) currently favours the Mayo score, or Disease Activity Index (DAI) [5], for trial design in ulcerative colitis, although it is not yet completely wedded to this. This brings arbitrary uniformity, but fails to bring objectivity, because the Mayo scoring system includes the highly subjective 'physician's global assessment'. Furthermore, the score includes a sigmoidoscopy subscore which is itself subjective, contributing additional variability and lack of precision. Also, the physician's global assessment takes into account the sigmoidoscopy score and is therefore not independent of the other elements.

There is in fact no reason to combine clinical, sigmoidoscopy, histopathology and quality of life variables into a single index. Indeed there is a strong argument against this. It is much easier to validate separate scoring systems for each component. Clinical trials can then be based on four validated scores, at least two of which (histology and quality of life) would usually be secondary endpoints. Indeed, even endoscopic mucosal healing could be a secondary endpoint, since this represents a tiny component (<1%) of complete remission, compared to subjective clinical remission recognised by the patient [19]. These endpoints remain, however, important to measure because they may influence long term outcome measures, such as the potential link between inflammation and carcinogenesis.

Despite careful evaluation of the strengths and weaknesses of all of these indices in the review of activity indices by authors from the International Organisation for Inflammatory Bowel Disease (IOIBD) [2], there is no escaping the fact that there has been no validation, nor any determination of inter-observer variability in scoring between indices. This has to be done. It is otherwise impossible to determine which index shows greatest consistency between observers, which matters enormously when investigators from four continents are recruiting patients to the same clinical trial. A practical example of the dilemma that this presents was the finding in 2006 that patients admitted from Russian centres to a clinical trial of a p38 MAP kinase inhibitor had significantly higher remission and response rates than non-Russian centres [20].

### Endoscopic scores

The general endoscopic grading system for ulcerative colitis was defined more than forty years ago by Baron et al [21]. It has been used in all trials of active ulcerative colitis to this day, with only minor (and unvalidated) modification [5]. The durability of this scoring system is astonishing; especially when it is considered that it was derived form observations made by 3 observers in 60 patients using rigid sigmoidoscopy. Nevertheless, the description and assessment of each component (compared to the unvalidated terms used by other indices, Table 3) means that it has largely stood the test of time. Four grades are defined (0–3) by the Baron score according to the severity of macroscopic inflammation of the rectal mucosal appearances at rigid sigmoidoscopy [Table 3]. The score has not been validated using flexible sigmoidoscopy and higher resolution endoscopes. Seven other endoscopic scoring systems have since been proposed, but none has gained similar acceptance [2].

Baron and two colleagues identified 14 visible variables that they scored and compared between observers [Table 4]. There was 40% disagreement on grading appearances as normal, mild, moderate, or severe activity. Not surprisingly, binary variables (present or absent) were associated with greater inter-observer agreement than graded variables. Unfortunately kappa scores are not available as the paper was written before the kappa statistic was widely applied to clinical medicine [22]. One variable, mucosal friability was pivotal in discriminating between mild and moderately active ulcerative colitis. This has acquired immediate clinical relevance now that common therapy has been shown to work for moderately active, but not mild, ulcerative colitis [23]. Friability in Baron's time was evaluated by wiping the mucosa with a cotton wool pledget on biopsy forceps or 'rocket swab' and seeing whether this provoked mucosal bleeding. The pressure needed and techniques were never defined, nor has this technique been validated in the era of flexible sigmoidoscopy when cotton wool pledges and rocket swabs are obsolete. Nevertheless, mucosal friability assessed at flexible sigmoidoscopy remains the pivotal discriminator not only for entry into clinical trials, but also for determining outcome. Patients who have no mucosal friability (Baron = 1 or 0) at outcome are deemed to have responded, as long as the mucosal friability (Baron = 2 or 3) was present at trial entry.

**Table 4:** Visible variables at sigmoidoscopy [21]

**Visible variable**	**Agreement (3 observers)**
• Spontaneous bleeding (present or absent)	
• Friability = 'bleeding to light touch' (present or absent)	
• Moisture (normal 'dry', moist, 'oedematous')	
• Distensibility (normal, rigid/contracted)	>60%
• Valves (normal sharp crescent folds, swollen, absent)	
• Large deep vessels (visible, not seen)	
• Ulcers (present or absent*)	
• Polyp (present or absent*)	

• Granularity (normal smoothness, granular)	
• Mucosal surface (attempt to describe the sheen on the mucosa: normal mat, dull lustreless; wet shiny)	<60%
• Colour (red, pink, pale)	
• Mucopus (no mucus or pus, clear mucus, opaque pus)	
• Faeces (none, solid, liquid)	
Small superficial vessels (normal, few, patchy)	

*:100% agreement, because none seen

Despite grading the severity of the appearance, the criteria did not claim any relation to disease severity. Baron et al observed that 'No attempt has been made to correlate these appearances with the clinical course or histological appearances' [21]. Remarkably, after 40 years, the score has still not been validated against clinical symptoms or histopathology of biopsy specimens. Nor has it been validated against outcome, although attempts have been made to correlate symptomatic (clinical) activity and endoscopic appearance (below). Furthermore, despite wide inter-observer variation and the pivotal role of endoscopy in clinical trials of ulcerative colitis [2,23-26], there has also been no attempt to determine intra-observer variation of scores using flexible sigmoidoscopy and digital imaging records viewed by the same observer on different occasions.

### Remission in ulcerative colitis

As if controversy about measuring disease activity was not enough, even disease remission has been neither defined nor validated. Remission is the outcome that matters in clinical trials, so agreement on the definition of remission is essential. Defining remission should logically be the starting point of agreeing how to measure activity in ulcerative colitis.

There are, however, at least three definitions of remission for ulcerative colitis. These may be termed clinical, registration and complete remission [Table 5]. Clinical remission is what is used in everyday clinical practice, meaning cessation of rectal bleeding and a normal stool frequency. This is not the same as 'registration' remission (the one currently, but not exclusively favoured by the FDA), which means cessation of rectal bleeding and a sigmoidoscopy score of 0 or 1 (equivalent to a normal appearance of the rectal mucosa, or erythema only [Table 3]). This, in turn is not the same as complete remission, which implies normal stool frequency, no rectal bleeding and a normal or quiescent appearances of the mucosa at sigmoidoscopy. The potential impact of these three definitions is considerable, but many trials simply use an arbitrary threshold to define the 'remission' endpoint. This is either 0, 1 or 2 of one of the disease activity indices, or <150 in the complex Seo index [2]. This variation makes it difficult to know what a trial means, because obscured in these low scores can be symptoms (such as bleeding or increased stool frequency) that clinicians and their patients would not recognize as remission. Because most trials choose different endpoints, let alone different activity indices, comparing the results of different trials is exceptionally difficult and the conduct of systematic reviews is seriously impeded.

**Table 5:** Definitions of remission in ulcerative colitis [40]

	**Characteristic**		
	**Stool frequency**	**Rectal bleeding**	**Sigmoidoscopy score**

**Clinical remission**	Normal	Absent	---
**Registration remission**	---	Absent	Normal or only erythema
**Complete remission**	Normal	Absent	Normal or only erythema

The Disease Activity Index (DAI, or Mayo score) is one of the most widely used of the activity indices in clinical trials. The impact of different definitions of remission using the DAI is illustrated by one large patient cohort. The ASCEND studies included a total of 687 patients with mild to moderately active ulcerative colitis, treated with 2.4 g or 4.8 g mesalazine [25,26]. Using these three different definitions of remission, the remission rate varied more than two-fold. When the DAI was 0, it was 22% (in other words, 'complete remission'); when the DAI was ≤ 1 the remission rate was 28% (meaning no bleeding and normal frequency, with at least a 1 point decrease in sigmoidoscopy score), but when 'remission' meant a DAI ≤ 2, it was 50% (meaning total score ≤ 2, with no individual subscore >1) [26]. This last definition of 'remission' is that used in the ACT trials of infliximab for ulcerative colitis refractory to standard therapy [27]. This is an extraordinary degree of variation; it is no wonder that doctors and patients are confused by different activity indices of clinical trials. When inter-observer variation in sigmoidoscopy scoring is factored in, the confusion becomes still greater.

### Inter-observer variation in sigmoidoscopy assessment

Inter-observer variation in sigmoidoscopy scoring is a crucial issue for regulatory authorities, since registration remission is based on just two components, sigmoidoscopy score and rectal bleeding. Investigators should expect variation between observers and expect that this variation is greatest when subjective assessments are made. What is required is that this variation is quantified. Recognition of this variation in clinical trials, furthermore, should lead both to training of observers in agreed standards and at least one additional observer when subjective assessments that are pivotal in regulatory terms (such as endoscopy) are being made. When an independent observer re-evaluated the sigmoidoscopy videos in a recent therapeutic trial of 335 patients with active ulcerative colitis, the observer disagreed with the investigators' sigmoidoscopy score in 12–23% of cases [28]. The impact on the remission rates of this variation in the sigmoidoscopy score was a median difference of 19% (range -10 to 22%) for absolute clinical, registration and complete remission. If results were then analysed according to the independent observer's score, remission rates were reduced in absolute terms by 10–16% for registration, but by <3% for clinical or complete remission. It is not surprising that registration remission rates were most affected. The implications are substantial. Inter-observer variation alone has the potential to make the difference between a therapeutically significant outcome and no response, and between licensed approval and no licence.

### Correlation between clinical activity and endoscopic mucosal appearance

It has been widely assumed that the activity of ulcerative colitis is related to the mucosal appearances seen at sigmoidoscopy. The concept is reasonable, but confidence is misplaced when sigmoidoscopic assessment is so subjective and clinical activity unvalidated [29]. When 222 observations of 10 symptoms and signs were compared with the sigmoidoscopic appearance, only the distinction between mucosa that bled spontaneously and that which bled on light touch or scraping was clinically meaningful in discriminating between moderate and severe disease [5]. Another study examined inter-observer agreement in the assessment of ulcerative colitis in 273 videotaped colonoscopies performed by 46 different endoscopists and then evaluated by two independent observers [30]. There was agreement on mucosal friability, spontaneous bleeding and mucopurulent exudates, which broadly correlated with clinical disease activity and histological activity scores. However, it has to be recognised that sigmoidoscopy contributes very little to complete remission (which includes symptomatic and endoscopic remission), compared to patient-defined remission (normal stool frequency, lack of urgency and bleeding) [19]. The different descriptive terms illustrate the need for minimum standard terminology for describing the mucosal appearance at sigmoidoscopy.

### Histology scores

Paradoxically the histological grading of ulcerative colitis has been subjected to the closest scrutiny [31-37], although histology is neither a criterion in any of the scoring systems, nor considered essential for the conduct of clinical trials by the FDA. Even so, eight separate scoring systems have been described for ulcerative colitis [2], although only three are widely used [[11,36] and [37]]. Inter-observer agreement has been assessed in a blinded fashion for 19 features. The features that provided most consistency (in distinguishing ulcerative colitis from Crohn's disease) were diffuse crypt architectural irregularity, general crypt epithelial polymorphs and reduced crypt numbers [35]. Binary variables (implying a 'yes' or 'no' answer, or ordered categorical variables) ensured the greatest agreement, as it was for endoscopy. Practical application of these variables was further tested between specialist gastrointestinal histopathologists, general histopathologists and trainees. Specialist histopathologists found location of neutrophils (in the lamina propria or between epithelial cells), the occurrence of crypt destruction, and the presence of erosions or ulcers to be the most consistent variables [36]. Once trained in identifying specific features, inter-observer variation between general histopathologists and trainees was similar (Kappa = 0.64 and 0.53 respectively). This implies that training is valuable, because it can both reduce interobserver variation and potentially reduce the need for specialist observers

Although histopathology of rectal biopsy specimens is not currently a trial requirement, there is a strong case for making it so. This is for reasons of diagnosis, safety and validation. Clinical trials in ulcerative colitis are recruiting centres in areas of the world (Eastern Europe, India, Russia, and South America) that are not widely recognised as having a clinical or research background in ulcerative colitis. Histopathology can corroborate the diagnosis, exclude infection (an important safety issue with biotherapy) and provide a permanent record. Trial validity is enhanced, because if a patient said to have active colitis actually has normal histology, then the diagnosis is wrong and the patient should not have been included in the trial. Furthermore, as mucosal healing emerges as a trial endpoint [38], histology provides independent corroboration.

### Unmet needs

There is consequently a pressing need to quantify inter-observer variation in videoendoscopic assessment of ulcerative colitis, and to study its relation to clinical symptoms and histopathology. The starting point should be to define the criteria for disease remission in a way that US and European drug regulatory authorities (FDA and EMEA) will recognise. Assessment of the degree of change in endoscopic score also needs to be quantified. Clinical trials have, until now, depended on unmatched scores at a single time point, rather than on paired assessments. This is because making a permanent endoscopic record has never been part of the procedure. Agreement on standard outcomes for clinical trials is fundamentally important. Such agreement can only be achieved by common consent among authorative bodies of experts (such as the International Organisation of Inflammatory Bowel Disease, IOIBD, or the European Crohn's and Colitis Organisation, ECCO), in conjunction with patient-perspectives. Such standard outcomes then have to be validated through clinical trials.

Apart from questions that matter to drug regulatory authorities, there are key questions of clinical relevance, which also affect the conduct and outcome measurement of trials [Table 6]. For instance, How often is there endoscopic activity when there is clinical remission? How often is the endoscopy normal when there is clinical disease activity? How often is there endoscopic activity when there is histological remission? and, How often is the endoscopy normal when there is histological activity.

**Table 6:** Unmet needs for outcomes of trials in ulcerative colitis

Develop a consensus definition of remission
*For each index of disease activity*:
Quantify the inter-observer variation for disease activity
Quantify the inter-observer variation for the degree of change between paired videos
Evaluate the relation between endoscopic score and clinical symptoms
Evaluate the relation between endoscopic activity and histological activity
Develop a consensus on a standard set of outcomes of disease activity to collect and report
Develop a consensus on a standard set of outcomes of clinical relevance to patients to collect and report (e.g. time to steroid-free remission, cumulative time off work or normal activities, hospital admission, colectomy)

### Potential solutions

To answer these questions demands a substantial resource of information, but this too can be defined:

1. Videoendoscopy of patients with active ulcerative colitis before and after treatment.

2. Standardisation of endoscopy preparation, procedure and assessment of mucosal friability.

3. Contemporaneous clinical scores on stool frequency and rectal bleeding.

4. Matching mucosal biopsies from pre-determined sites.

The process would necessitate a random selection of videoendoscopies to be scored according to pre-determined criteria evolving from Table 2, by a group of acknowledged authorities in endoscopic and clinical practice, so that a kappa statistic with narrow confidence interval can be calculated for each component. Pre- and post-treatment videoendoscopies would be randomised to avoid explicit pairing and allow consistency to be evaluated, as well as the ability to determine the degree of change between videos from the same individual. The group of features with the least inter-observer variation would then be available for a validated scoring system. This need not be limited by the graded terms mild, moderate, or severe, but could simply define features such as mucosal friability, spontaneous bleeding or mucosal ulceration. Correlation between endoscopic appearance, clinical features and histology is then possible.

Such a resource exists as a consequence of a recent clinical trial on ulcerative colitis. (EUDRACT no: 2004-004077-29). There are 670 videoendoscopies available for review (paired videos on each of 335 patients). These endoscopies were performed by experienced endoscopists who received specific training on the conduct of procedures for the study, and this training was reinforced when the independent observer disagreed with the investigator's sigmoidoscopy score during the trial. The criteria for the conduct of the procedure, including preparation, technique of eliciting mucosal friability, and biopsy sites were pre-determined. Plans are in place to select videoendoscopies for analysis by a group of experts in endoscopy and the clinical management of ulcerative colitis and scoring them independently, to define a kappa statistic with narrow confidence intervals. Endoscopic scores can then be related to contemporaneous data on stool frequency, rectal bleeding, and histopathology. Once such data are available then it would be appropriate to develop a standard set of outcomes for randomised controlled trials in ulcerative colitis.

## Conclusion

In trials of ulcerative colitis the lack of validated activity indices, the lack of an internationally agreed definition of remission, and the failure to quantify inter-observer variation in sigmoidoscopy scoring has a major impact on outcome that is largely unrecognised. This affects registration of new drugs and makes meaningful comparison between clinical trials exceptionally difficult. There is now an opportunity to address inter-observer variation in sigmoidoscopy scoring and to relate results to clinical activity and histopathology scoring. This will help set standards for clinical, endoscopic, and histopathological data collection and interpretation for future clinical trials in ulcerative colitis allowing comparison and combination of trial results as is the case in rheumatology. It will facilitate the international training of endoscopists in the appearance and description at endoscopy. Once established it can be used to assist regulatory evaluation of evolving therapies and in national audit. Separate, validated scoring systems for clinical activity, sigmoidoscopy, histopathology and quality of life, rather than a composite index, appear fundamental to clinical trial design. The ultimate goal is to reach a consensus on key outcomes that would always be assessed and reported in clinical trials.

## Competing interests

SPLT has acted as adviser to, lectured for and received unrestricted educational grants or research support from Abbott, Ferring, Procter & Gamble, Schering Plough, Shire, UCB.

Other authors: None declared

## Authors' contributions

RMC: Drafting and revisions of script

BFW: Drafting of histology section and critical review of paper

MTA: Participated in design of paper and critically reviewed paper

DGA : Participated in design of paper and critically reviewed paper

SPLT: Conception of paper, drafting and editing of script

All authors read and approved the final manuscript.
